# Extraneural Metastasis of Primary Glioma Occurring in a Setting of Occupational Ionizing Radiation Exposure

**DOI:** 10.1155/2019/1748739

**Published:** 2019-06-13

**Authors:** Nitya Prabhakaran, Douglas C. Miller, N. Scott Litofsky, Shellaine R. Frazier

**Affiliations:** ^1^Department of Pathology and Anatomical Sciences, University of Missouri School of Medicine, Columbia, MO 65212, USA; ^2^Division of Neurological Surgery, Department of Surgery, University of Missouri School of Medicine, Columbia, MO 65212, USA

## Abstract

Malignant gliomas account for 60% of all primary brain tumors in adults. Glioblastoma Multiforme (GBM) is the most common primary glial tumor with a dismal prognosis and a median survival of approximately 14 months. Extra-neural metastases from primary brain tumors are unusual with an incidence rate of less than 2%. This has been attributed to factors such as short survival, lack of true lymphatics in the CNS, and physical barriers provided by the dura, extracellular matrix, and basement membrane. Although most GBMs occur sporadically, there is a known association with therapeutic radiation exposure and with work in nuclear disaster cleanup. To our knowledge, no case of GBM with metastasis occurring in a patient with occupational radiation exposure currently exists in the literature. In this article, we present a case of GBM with lung metastasis occurring in a 51-year-old Caucasian male, whose history is significant for occupational exposure to ionizing radiation, and review the literature on GBM risk factors and potential mechanisms of metastasis.

## 1. Introduction

Glioblastomas (GBMs) are the most common and yet the most aggressive primary intraparenchymal brain tumors in adults [1–9]. They occur throughout the central nervous system (CNS) but are most common in the brain [1, 5]. Primary malignant brain tumors are thought to develop through accumulations of genetic alterations that permit cells to evade normal regulatory mechanisms and escape destruction by the immune system [1]. Factors that have positive and negative associations with GBM development have been described. While allergies or atopic disease(s) have been reported by some to decrease the risk, an increased risk has been shown with exposure to ionizing radiation (IR) [3, 10, 11]. An association with high-dose IR and all brain tumors has been observed in atomic bomb survivor studies, nuclear-test fallout data, therapeutic radiation for cancer or for benign conditions, and occupational and environmental studies [1, 10–12]. Like all primary brain tumors, extraneural metastasis of GBM is very rare, reported in less than 2% of all GBM cases [4, 7–9, 13–19]. The exact mechanisms of spread outside of the CNS of gliomas are not completely understood. The rarity of this phenomenon has been attributed to the usually short survival of affected patients and also due to intrinsic biological obstacles that prevent the tumor cells from infiltrating and surviving beyond the CNS environment [4, 7–9, 13–19]. Furthermore, we present a unique case of a middle-aged Caucasian man with a history of occupational IR exposure who developed a GBM with subsequent metastasis to the left lung.

## 2. Material and Methods

### 2.1. Presentation and Clinical Course

**Figure 1:**
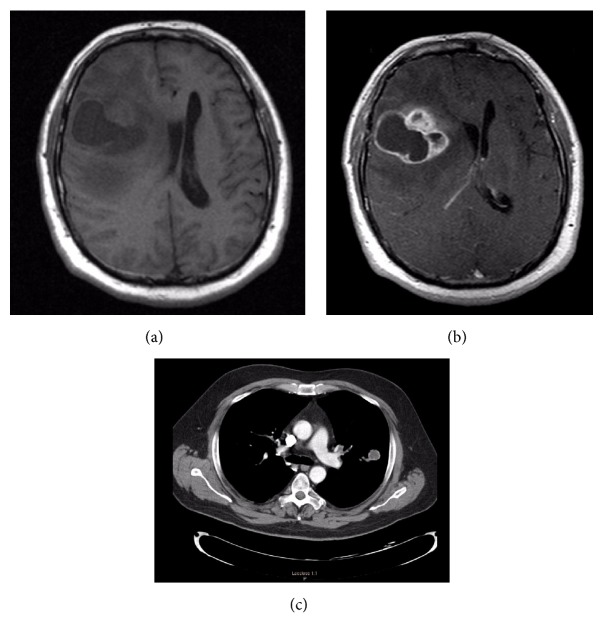
(a) and (b): pre- and postcontrast MRI demonstrating large right frontal ring-enhancing mass, October 2008. (c) Chest CT with mass in the left lung, May 2009.

A 50-year-old Caucasian man nuclear power plant worker with an unremarkable medical history presented with complaints of recurrent headaches, nausea, and vomiting with left sided facial droop and personality changes, all occurring for 3-4 weeks. A computerized tomography (CT) scan of the brain showed a right frontal mass. Subsequently a pre- and postcontrast magnetic resonance imaging (MRI) was performed that showed a right frontal mass [Figures 1(a) and 1(b)]. A right frontal craniotomy with gross total excision of the tumor was performed, after which the patient was treated with chemoradiation. Despite this, the patient continued to have frequent headaches and tremor with occasional gait imbalance. He also suffered multiple episodes of abdominal pain and diarrhea that was attributed to increased dosage of Temozolomide. The patient came back with increased frequency of headaches. This prompted a magnetic resonance imaging (MRI) scan which showed a large recurrent tumor. He subsequently underwent a right frontotemporal craniotomy with gross total excision of the recurrent mass, followed by Temozolomide and radiation therapy. Six months later, chest imaging performed in preparation for insertion of a central catheter showed a left upper lobe lung lesion which was suspicious for malignancy [Figure 1(c)]. This was removed as a wedge resection and was found to contain a malignant tumor which was morphologically and immunohistochemically identical to the primary brain tumor. The patient died 16 months after his initial presentation.

### 2.2. Pathology Findings

**Figure 2:**
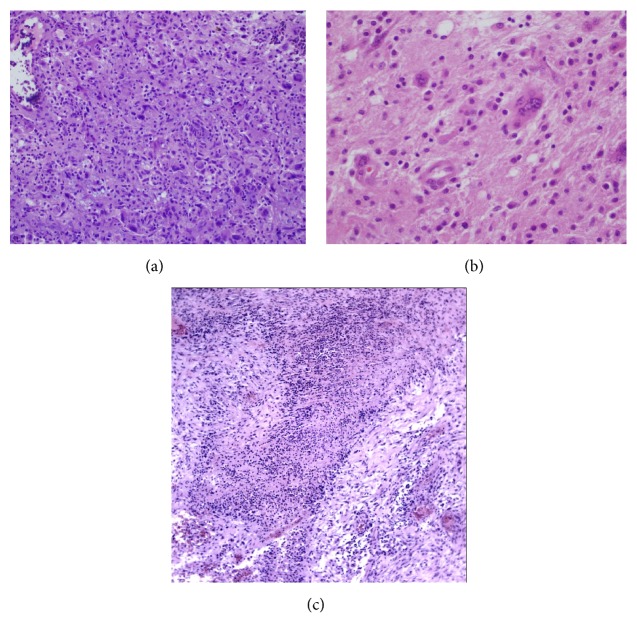
Brain tumor. (a) The tumor is composed of large and small cells (LFB/H&E, 100x). (b) The tumor contains many multinucleated giant cells (LFB/H&E, 400x). (c) The tumor displaying necrosis with palisade (H&E, 100x).

Histologically, the initial resection specimen of the right frontal lobe mass was a high-grade glioma that infiltrated the adjacent brain tissue [Figure 2]. The tumor had combined features of a high-grade glioma, WHO grade IV. There were foci of necrosis surrounded by palisades of tumor cells, extensive vascular hyperplasia, and a high mitotic rate. The tumor was composed of a mixture of small to medium sized cells and some very large, often multinucleated, giant cells [Figures 2(a)–2(c)]. The cytoplasm of the large cells was brightly eosinophilic. The background of smaller cells included a mixture of cells with round nuclei centrally placed in clear to pale cell bodies. Immunostains revealed nearly 100% tumor cell immunopositivity for vimentin, with the large cell population also immunopositive for glial fibrillary acidic protein (GFAP). The smaller cell population was negative for GFAP. An immunostain for Ki-67 showed up to 50% tumor cell nuclear immunopositivity in the most labeled areas. An immunostain for the protein product of the isocitrate dehydrogenase-1 R132H mutation (IDH1 R132H) showed no immunoreactivity in tumor cells.

The resection of the recurrent brain tumor yielded a high-grade infiltrating glioma with predominantly astrocytic features in that the predominant cell population had elongated bipolar processes. The tumor again had vascular hyperplasia. There was also extensive radiation induced necrosis.

**Figure 3:**
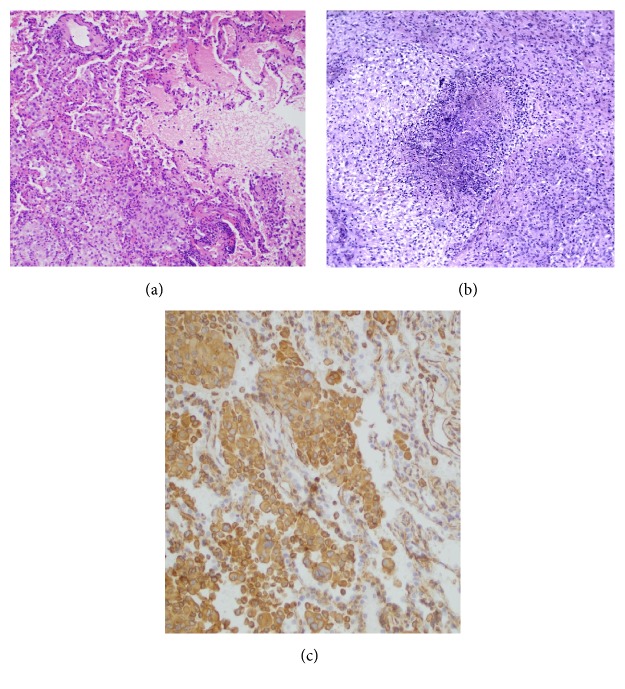
Lung tumor. (a) The tumor forms a solid mass (left side) with bordering alveoli (right side) (H&E, 100x). (b) Necrosis in the lung tumor (H&E, 100x). (c) GFAP immunostain demonstrating immunopositive tumor cells in the lung with immunonegative pulmonary cells (right side) (GFAP immunostain, 100x).

The left lower lobe lung tumor was a metastatic glioma characterized by nests and sheets of cells with moderate to abundant amounts of eosinophilic to pale cytoplasm [Figure 3(a)]. Individual cells had eccentrically placed nuclei with moderate to marked pleomorphism. The markedly enlarged and hyperchromatic nuclei had prominent nucleoli, and, occasionally, nuclear pseudoinclusions. Mitotic figures were easily identifiable, and there were areas of necrosis [Figure 3(b)]. Immunostains revealed tumor cells with cytoplasmic immunoreactivity for vimentin, GFAP [Figure 3(c)], and S100 protein along with rare cells immunopositive for epithelial membrane antigen (EMA). These EMA-immunopositive cells were consistent with entrapped normal lung epithelial cells. There was no immunoreactivity for cytokeratin (CK), epithelial membrane antigen (EMA), thyroid transcription factor 1 (TTF1), synaptophysin, HMB45, or Melan A/MART1 (antibody A103) in the neoplastic cells.

## 3. Discussion

Historically, the glial origin of GBMs was first recognized by Virchow in 1863. The erstwhile term “glioblastoma multiforme” was coined by Mallory in 1914 [2]. In 1926 Bailey and Cushing definitively changed the name from spongioblastoma multiforme to GBM and also went on to state that distant metastases from primary brain tumors do not occur [2, 7, 19, 20]. Scherer and Kernohan each recognized that GBMs may sometimes develop from the progression of a low-grade lesion, an observation that has since been confirmed by molecular studies [2]. The first case of extraneural metastases from a GBM was reported in 1928. In 1955, Weiss suggested diagnostic criteria for extraneural metastases from primary CNS tumors, which included a documented history of a primary CNS tumor, and histologic similarity between the primary tumor and presumed metastatic lesion [9]. More recently immunohistochemical similarities have also been important in such diagnoses.

Incidence rates of gliomas vary significantly by histologic type, age at diagnosis, sex, race, and country [1, 3, 10, 11]. Many factors have been postulated as potential risk factors for gliomas, including both heritable and acquired factors. Although numerous familial cancer syndromes are associated with an increased glioma risk, monogenic Mendelian disorders account for only a small proportion of adult glioma incidence at the population level [1].

Several epidemiologic risk factors have also been examined as potential contributors to glioma risk. Amongst these factors, those which seem to have a significant association include a decrease in risk by history of allergies or atopic disease(s) and an increase in risk by exposure to IR [1, 3, 10, 11].

Certain forms and doses of IR are generally accepted as having the potential to cause brain tumors. Atomic bomb studies, nuclear test fall-out data, studies of survivors of therapeutic radiation for cancer and for benign conditions, and occupational and environmental studies have connected IR to glioma genesis [11]. Nuclear facility employees and nuclear material production workers may have an elevated relative risk for brain tumors. Loomis and Wolf followed workers exposed to nuclear radiation between 1947 and 1974. They found that deaths from brain cancer and lymphopoietic malignancies were increased in white men so exposed [20]. Though several studies have been done in the past to demonstrate a heightened risk of development of glioma in patients exposed to IR [21–28], conflicting opinions exist in the scientific literature.

The data showing the association between radiation and gliomas is limited but data in adults show heightened greater risk in certain groups exposed to radiation. Two cohorts from Latvia and Estonia that consisted of approximately 10,000 individuals exposed to nuclear radiation showed a statistically significant increase in brain cancers [29]. Cardis* et al*. reviewed 95,673 workers who were exposed to IR for six months or longer across three countries. They found that mortality from leukemia, excluding chronic lymphocytic leukemia (CLL), was most strongly and consistently related to low dose IR exposure [30]. In a review of ten carefully conducted cohort mortality studies of U.S. workers in the nuclear industry published during the past decade, Alexander mentions that there is a significantly increased brain cancer risk. Further, these index studies of more than 78,000 workers followed for an average of 21 years, with more than 1.6 million person years of observation, establish that there is a statistically significant 15% increased risk of brain cancer for workers in the U.S. nuclear industry who have low-dose average cumulative radiation exposures [31]. Our patient had a history of working as a reactor operator in a nuclear power plant. The duration and dose of exposure were not available which makes it difficult to assess whether the IR exposure could have been a contributory factor in his GBM.

Morphologically GBMs are composed of cells that resemble astrocytes with irregular, ovoid, or elongated but mostly round nuclei that tend to lie toward one side or edge of the cell body. The* sine qua non* for this diagnosis is tumor necrosis. Often this necrosis is surrounded or outlined by a “palisade” of small poorly differentiated tumor cells with negligible eosinophilic cytoplasm and elongate hyperchromatic nuclei. Other features include vascular hyperplasia, mitotic figures in tumor cells, and pleomorphism [32].

Metastatic spread of GBM outside the CNS is unusual, reported to occur in less than 2% of all GBMs [4, 7–9, 13–19]. Pasquier* et al*. reviewed the reported cases of extraneural spread of GBM and documented 72 case reports published between 1928 and 1980 [33].

The most common sites of metastatic spread are the lungs and pleurae followed by lymph nodes, bone, and liver [34]. Patients with lung metastases are known to have the worst prognosis [13].

Several theories have been proposed as possible routes and mechanisms of such metastatic spread but, despite all the theories and evidence, such metastasis by gliomas is still a poorly understood process. Historically, it has been postulated that the lack of lymphatic vessels in the CNS constitutes a barrier to tumor spread [4, 7, 8, 13, 15, 18, 19]. However studies done in the last few years have highlighted the presence of a robust surrogate for a lymphatic system within the CNS.

Functional lymphatic vessels lining the dural sinuses have been demonstrated. These structures express all the molecular hallmarks of lymphatic endothelial cells (LYVE1, PROX1, and vascular endothelial growth factor receptor 3 (VEGFR3)) and are able to carry both fluid and immune cells from the CSF and drain to the deep cervical lymph nodes [35, 36]. Two types of afferent lymphatic vessels are known to exist. These are of the initial and collecting types. They differ anatomically in the expression pattern of adhesion molecules and in their permissiveness to fluid and cell entry. In contrast to the dural venous sinuses, the meningeal lymphatic vessels are devoid of smooth muscle cells. In addition, the meningeal lymphatic vessels are immunopositive for the immune-cell chemoattractant protein, CCL21 [35].

In 2014, a study done by Muller* et al*. identified the presence of circulating tumor cells (CTC) in the peripheral blood in 29 of 141 patients with GBM by immunostaining of enriched mononuclear cells with antibodies directed against GFAP, thus demonstrating hematogenous dissemination of glioma cells [15]. The concept of CTCs has also been mentioned in a few other studies [8, 13, 14]. The hematogenous route is the main pathway for metastases to lung, bone, and spleen [7]. In 2014, Ray* et al*. reviewed three case reports of systemic metastasis of GBMs and stated that the majority of metastases occur through the leptomeninges or intramedullary dissemination to the spinal cord [4].

Patients with GBM typically respond initially to therapy but ultimately relapse within the high-dose irradiation field, suggesting the presence of a subpopulation of resistant cells. While heterogeneity of tumors in different patients can in part explain varied patient responses to therapy,* intratumoral* heterogeneity is now recognized as a critical factor in determining therapeutic response. GBM initiating cells (GICs) are a subgroup of cancer stem cells that exhibit the ability to self-renew and express putative stem cell markers such as CD133, SSEA-1 (CD15), L1CAM, and CD44. GICs are defined functionally by their ability to repopulate the tumor upon serial transplantation [6].

In 2000, Park* et al*. reported the molecular genetic findings in six cases of extraneural metastasis of GBM. Four of these cases had TP53 mutations, but remarkably two different TP53 alterations were observed in paired primary and metastatic tumors [37]. This would suggest the emergence of subclones which were not dominant in the original GBM arising in the brain [4, 7]. A meta-analysis done by Elena* et al*. supports the idea that prolonged survival of GBM patients is associated with a greater risk of extraneural dissemination [14].

The standard therapy for newly diagnosed GBM is the “Stupp regimen” that uses Temozolomide and radiation. Our patient failed this regimen and in fact developed a metastatic lesion that we have described in this case report [38].

## 4. Conclusion

While gliomas occurring in the setting of occupational IR exposure may not represent a distinct clinical entity, it has been shown that certain forms of IR exposure do impart an increased risk of GBM development. The occurrence of a metastatic glioma in our patient with a history of occupational radiation exposure is both unique and intriguing as to what role might have been played by his occupational IR exposure. If the dose and duration of exposure were known in our patient it would have contributed to a better understanding of the association between low dose and high dose IR with primary brain tumors such as gliomas. This perhaps warrants more focused studies exploring the effects of IR on specific tumor subtypes and their relationship to specific dosage and duration of exposure. As innovative therapeutic interventions such as targeted therapies become increasingly available, longer GBM survival rates and subsequently more cases of dissemination of GBMs outside the CNS can be expected. For this reason, it is essential that physicians be cognizant of the potential for such metastatic disease in patients with GBMs.
